# Harnessing Repurposed Drugs to Enhance Temozolomide Efficacy in Glioblastoma

**DOI:** 10.1002/cnr2.70643

**Published:** 2026-08-05

**Authors:** Ali Nakhaei, Atefeh Taghavi, Amir R. Afshari, Farzaneh Davoudi, Elaheh Gheybi, Mohammad Jalili‐Nik

**Affiliations:** ^1^ Student Research Committee, MMS.C Islamic Azad University Mashhad Iran; ^2^ Department of Basic Sciences, Faculty of Medicine, Mashhad Medical Sciences Islamic Azad University Mashhad Iran; ^3^ Bahman Educational and Medical Charity Hospital Mashhad Iran; ^4^ Department of Clinical Biochemistry, Faculty of Medicine Mashhad University of Medical Sciences Mashhad Iran; ^5^ Department of Biology, School of Computer, Mathematical, and Natural Sciences Morgan State University Baltimore Maryland USA; ^6^ Pharmacological Research Center of Medicinal Plants, Basic Science Research Institute Mashhad University of Medical Sciences Mashhad Iran

**Keywords:** chemotherapy resistance, drug repurposing, FDA‐approved, glioblastoma, Temozolomide

## Abstract

**Background:**

Glioblastoma (GB) is the most aggressive primary malignant brain tumor in adults and remains associated with poor survival despite surgical resection followed by radiotherapy and temozolomide (TMZ) chemotherapy. Intrinsic and acquired resistance to TMZ, including MGMT‐dependent DNA repair and activation of pro‐survival pathways, could decrease treatment efficacy. Drug repurposing offers an attractive strategy to identify agents that may enhance TMZ activity because these drugs already have known pharmacokinetic and safety profiles. This narrative review summarizes the available evidence on repurposed drugs investigated as potential modulators of TMZ response in GB.

**Recent Findings:**

A range of repurposed agents, including chloroquine, valproic acid, levetiracetam, metformin, aspirin, amlodipine, atorvastatin, chlorpromazine, melatonin, disulfiram, bortezomib, and verteporfin, have been evaluated in GB models and selected clinical studies. Reported mechanisms include modulation of MGMT expression, autophagy, oxidative stress, apoptosis, DNA‐damage responses, cancer stem‐cell properties, and signaling pathways such as PI3K/AKT/mTOR, AMPK, EGFR, STAT3, ERK1/2, and NF‐κB. Several agents have enhanced TMZ‐associated cytotoxicity in cell culture and animal models. However, clinical evidence remains limited, and the results are inconsistent for some drugs. Blood–brain barrier penetration, achievable intratumoral drug exposure, toxicity, treatment scheduling, and molecular heterogeneity of GB remain major translational challenges.

**Conclusion:**

Repurposed drugs may provide useful candidates for improving TMZ‐based therapy in GB. However, most evidence remains preclinical, and further studies are needed to clarify blood–brain barrier penetration, optimal dosing, toxicity, predictive biomarkers, and clinical efficacy. Well‐designed prospective clinical trials are required before these combinations can be incorporated into routine GB treatment.

## 1 Introduction

Glioblastoma (GB) represents the most prevalent and aggressive primary malignant brain tumor in adults. The current standard therapeutic strategy for newly diagnosed GB includes surgical resection followed by radiotherapy in combination with chemotherapeutic agents such as temozolomide (TMZ) [1]. TMZ exerts its anti‐tumor effect by adding methyl groups to DNA, most importantly at the O^6^ position of guanine. This specific lesion mispairs during DNA replication, triggering DNA damage responses that ultimately lead to tumor cell death. However, glioblastoma cells can counteract this effect through the DNA repair enzyme O^6^‐methylguanine‐DNA methyltransferase (MGMT), which directly removes the methyl group and restores the DNA, thereby reducing the drug's cytotoxicity [2].

Drug repurposing, the process of finding new therapeutic uses for existing drugs, has many advantages over developing new drugs. It allows drugs to reach the clinic more quickly, as their safety and pharmacokinetic data are already available, reducing the need for initial testing. It also reduces research and development costs and may uncover new molecular targets and biological insights that expand the therapeutic potential of existing drugs [3, 4].

**FIGURE 1:**
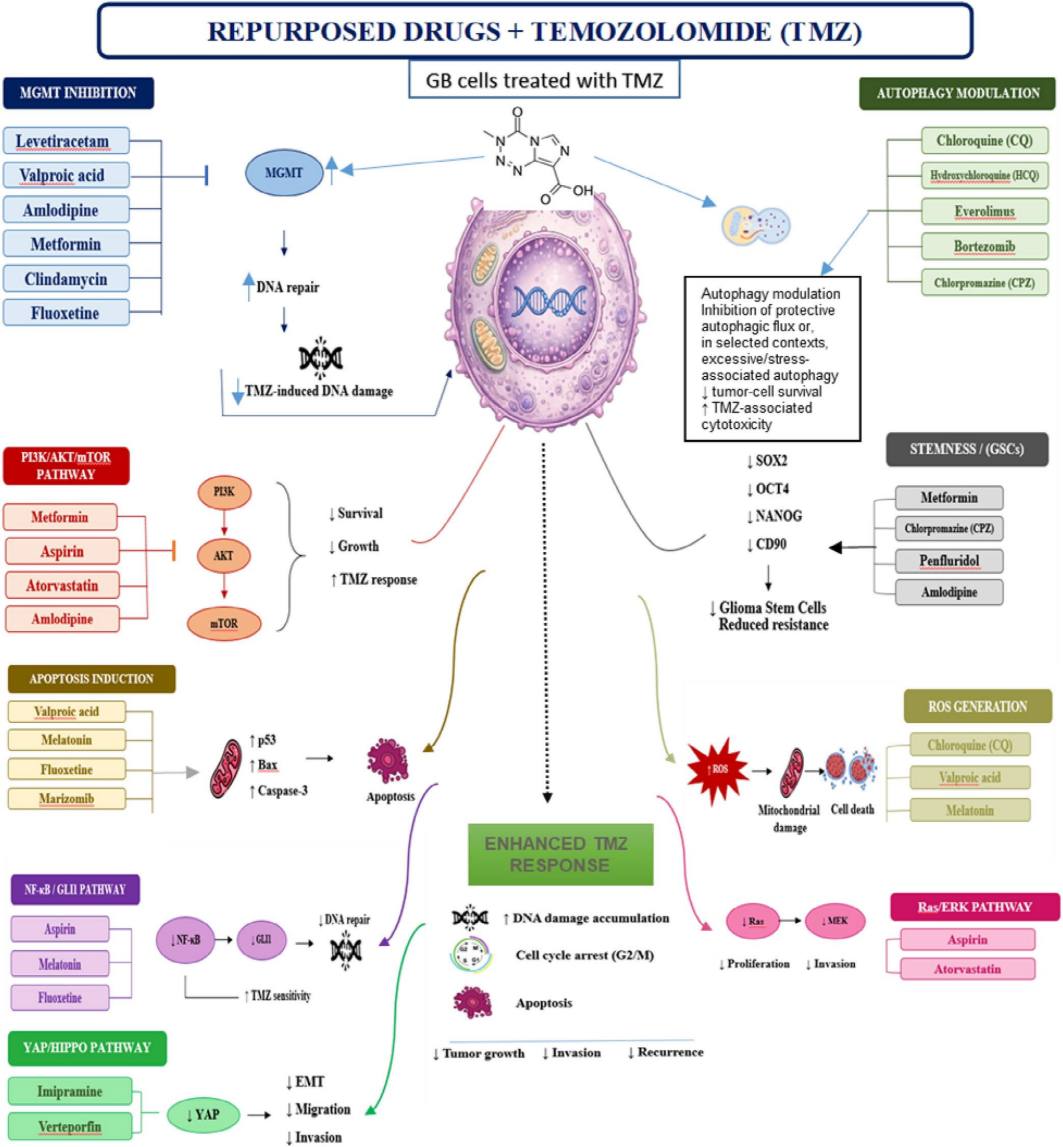
Proposed molecular mechanisms through which repurposed drugs may enhance temozolomide (TMZ) response in glioblastoma. Candidate drugs may modulate MGMT‐mediated DNA repair, autophagy, apoptosis, oxidative stress, PI3K/AKT/mTOR, NF‐κB/GLI, Ras/ERK, YAP/Hippo, and glioma stem‐cell pathways. Most findings are preclinical, and clinical benefit requires further validation.

This review describes the therapeutic mechanisms and targeted pathways of repurposed drugs used in combination with TMZ, their mode of action, and their potential clinical applications for GB. This narrative review discusses agents that are approved for other indications in one or more jurisdictions, together with selected investigational agents that have been proposed as TMZ modulators, including: antimicrobials (e.g., chloroquine), central nervous system drugs (e.g., valproic acid), cardiovascular drugs (e.g., aspirin, amlodipine), antidiabetic drugs (e.g., metformin), lipid‐lowering drugs (e.g., atorvastatin), antipsychotics (e.g., chlorpromazine), opioids (e.g., methadone, morphine), antibiotics (e.g., nitroxoline, azithromycin, ciprofloxacin), benzodiazepines (e.g., diazepam), and SSRIs (e.g., fluoxetine), supplements (e.g., melatonin), hormone therapy (e.g., tamoxifen), anti‐alcoholism agents (e.g., disulfiram), proteasome inhibitors (e.g., marizomib, bortezomib), mTOR inhibitors (e.g., everolimus), and photodynamic therapy agents (e.g., verteporfin). This narrative review was based on a literature search of PubMed and Web of Science. Search terms included glioblastoma, glioma, temozolomide, drug repurposing, and the names of individual candidate drugs. Original preclinical and clinical studies evaluating repurposed drugs alone or in combination with temozolomide were included. Review articles were used to identify relevant primary studies. Studies unrelated to glioblastoma or lacking relevant outcome data were excluded. Preclinical and clinical findings were evaluated separately. These drugs may modulate key pathways involved in glioblastoma growth, survival, DNA repair, autophagy, and TMZ resistance, including MGMT, PI3K/AKT/mTOR, AMPK, EGFR, STAT3, ERK1/2, NF‐κB, and YAP/Hippo signaling pathways (Figure 1; Table 1). Furthermore, this review demonstrates the clinical importance of repurposed drugs and offers the potential to provide more effective and accessible treatment options for this highly aggressive tumor type.

**TABLE 1:** Preclinical evidence for repurposed drugs in combination with temozolomide in glioblastoma models.

Author	Drug	Type of study	Effect	References
Lee et al.	Chloroquine	U87 cell	↑autophagic vacuoles ↑p53 and phospho‐p53 levels no enhanced effect on U373 cell (mutant p53) proliferation and apoptosis ↓clonal and cellular growth with increased G_2_–M arrest	[5]
Hori et al.	Chloroquine	Rat C6 glioma cells and human U87‐MG GB cells	↑mitochondrial ROS and cell death ↑colocalization of EGFP‐LC3 dots with mitochondria ↑cytotoxicity in patient‐derived GB cells as well as human U87‐MG GB cells	[6]
Ryu et al.	Valproic acid	U87, U138, T98, and U251 cells	↑antitumor effect in TMZ‐resistant malignant glioma cells (T98 and U138) ↑apoptotic and autophagic cell death ↓the migratory activities in TMZ‐resistant cell lines ↓tumor growth compared with the monotherapy groups of mice.	[7]
Chen et al.	Valproic acid	The human glioma cell lines, U87MG, Hs683, and DBTRG‐05MG	↑apoptosis in U87MG cells as evidenced by p53 and Bax expression, mitochondrial transmembrane potential loss, ROS production, and glutathione depletion ↓nuclear translocation of the nuclear factor‐erythroid 2 p45‐related factor ↓heme oxygenase‐1 and γ‐glutamylcysteine synthetase expression.	[8]
Tsai et al.	Valproic acid	The GB cell lines U87, DBTRG‐05MG, U118MG, and LN229	↑antineoplastic effect of TMZ by enhancing p53 activation and promoting the expression of its downstream proapoptotic protein, PUMA GB patients with wild‐type *p53* may benefit from a combined TMZ + VPA treatment.	[9]
Chen et al.	Valproic acid	The human GB cell line U87MG, DBTRG‐05MG, and M059K / 5‐week‐old female athymic nude mice	AR may potentially serve as biomarker and therapeutic approach for chemotherapy regimens in GB.	[10]
Navone et al.	Aspirin	Human primary GB–endothelial cells (ECs)	Regulated GB‐EC viability, tube‐like structure formation, cell migration, and VEGF releasing ↓VEGF, VEGFR‐1, HIF‐1α, RAS, mitogen‐activated protein kinase kinase, AKT, and BCL‐2	[11]
Ming et al.	Aspirin	The human glioma cell lines, U87 and T98G	↓SHH/GLI1 pathway and reprogramming the EMT. ↓nuclear translocation of GLI1 to inhibit its transcriptional regulation. ↑chemosensitivity and GLI1‐induced chemoprotection was partly blocked by ASA In vitro and In vivo.	[12]
Feng et al.	Metformin	GB cell lines U87MG, LNZ308, and LN229	↓cell viability, proliferation, and migration ↑cell apoptosis disrupt EMT in all 3 GB cell lines ↑ROS production, impaired mitochondrial membrane potential ↓mitochondrial transcription factor A, Twist, and MGMT proteins in TMZ‐resistant LN229 cells	[13]
Valtorta et al.	Metformin	The human U251 and T98G GB cell lines	modulated apoptosis by increasing Bax/Bcl‐2 ratio, and reduced ROS production ↓expression of GSC marker CD90 particularly in T98G cells slowed down growth of TMZ‐resistant tumors but did not affect OS of TMZ‐sensitive tumor bearing mice.	[14]
Yang et al.	Metformin	Human GB cell lines U‐87MG and U‐251MG	down‐regulates SOX2 expression in TMZ‐resistant glioma cells ↓neurosphere formation capacity of GB cells ↓GB xenograft growth in vivo.	[15]
Yu et al.	Metformin	The human glioma cell lines U87 and U251	↓AKT activation induced by TMZ ↓mTOR, 4EBP1 and S6K phosphorylation. ↓tumor growth rates and ↑median survival of tumor‐bearing mice.	[16]
Peng et al.	Atorvastatin	Four human GB cell lines A172, T98G, U87 and U251	↓growth and survival of multiple GB cell lines. ↑TMZ's efficacy in in vitro cultured cell system as well as in vivo xenograft GB tumor model. this is achieved by the inhibition of Ras prenylation, leading to decreased activation of Ras and its downstream signaling pathways, including Erk, rS6 and eIF4E.	[17]
Bobustuc et al.	Levetiracetam	Hs683, SW1088, SW1783, U87, A‐172, LN18, T98G, and U138 human glioma cells	↓MGMT protein and mRNA expression levels. ↑p53 binding on the MGMT promoter by recruiting the HDAC1 corepressor complex. ↓malignant glioma cell proliferation ↑glioma cell sensitivity to the monofunctional alkylating agent TMZ.	[18]
Scicchitano et al.	Levetiracetam	A total of six pairs of neurospheres derived from both GB and peritumoral tissue called GCSCs and PCSCs	↑the effect of TMZ on GCSCs proliferation (being less effective on PCSCs) by decreasing MGMT expression, promoting HDAC4 nuclear translocation and activating apoptotic pathway	[19]
Ni et al.	Levetiracetam	Human glioma cell lines U138, U118, T98 G, SKMG‐4, U87, U373, and U251 / Four‐week‐old female BALB/c‐nu/nu mice	The combination use of LEV and IFN‐α could be an optimal method to overcome TMZ resistance through obvious MGMT inhibition in MGMT‐positive glioma.	[20]
Marutani et al.	Levetiracetam	Human glioma cell lines T98 and A172	LEV has a tumor‐inhibiting effect in GB cell lines, which was enhanced when treated in combination with TMZ. LEV could therefore be used in conjunction with TMZ, which would enable reduction of the TMZ dose to achieve an improved tumor response and reduced side effects.	[21]
Zhang et al.	Amlodipine	The human GB cell line T98G	↓the GSC viability. ↑apoptosis, cell cycle arrest in vitro. ↓tumor volume. ↑the cytotoxicity of TMZ against GSCs by downregulating MGMT expression through the suppression of the Akt/GSK3β/β‐catenin axis.	[22]
Matteoni et al.	Chlorpromazine	Anchorage‐dependent cell lines T98G, U‐251 MG and U‐87 MG	↓viability in an apoptosis‐independent way, ↑hyperdiploidy, ↓cloning efficiency as well as neurosphere formation and ↓expression of stemness genes in all these cell lines.	[23]
Matarrese et al.	Chlorpromazine	Anchorage‐dependent GB cell lines U‐87 MG and U‐251 MG, as well as hTERT‐immortalized human retinal pigment epithelial cells hTERT RPE‐1	↓connexin‐43 expression in GB cells, consequently compromising connexin‐dependent cellular resilience, and ultimately contributing to cell death.	[24]
Bostanci et al.	Melatonin	C6 ( *Rattus norvegicus* ) and N1E‐115 ( *Mus musculus* ) cancer cell lines and C8‐D1A (mice) healthy cell lines	↓the viability of both GB and neuroblastoma cells compared to the vehicle‐treated controls. ↑genes of antioxidative enzymes (Sod1 and Sod2) and DNA repair genes (Mlh1, Exo1, and Rad18) in both cell lines. ↓levels of Nfkb1 and Pik3cg ↑cell cycle arrest and increased the expression of p53 and proapoptotic proteins (Bax and caspase‐3)	[25]
Tang et al.	Melatonin	The human GB cell lines U87‐MG and U118‐MG, as well as the murine glioma cell line GL261	↓ GB cell proliferation, migration, and invasion. Regulated NF‐κB/COX‐2 signaling pathway. ↑TMZ's antitumor activity by inhibiting IκBα phosphorylation, suppressing NF‐κB activation, and downregulating COX‐2 expression. ↑apoptosis via activation of the Caspase‐3 pathway.	[26]
Gil et al.	Melatonin	GB patient‐derived cell lines were treated with melatonin at 1.5 or 3 mM alone or in combination with TMZ 0.7 or 3 mM for 48 h.	Disrupting BTIC metabolism with MEL leads to cell dead and by chemosensitizing them, it would allow a reduction in TMZ dosage, therefore decreasing possible side‐effects and indicating MEL as a strong therapeutic candidate for GB therapy.	[27]
Wang et al.	Imipramine	Glioma cell lines (U87, U251, U373, LN229, GL261) and normal human astrocyte (NHA)	↓tumor proliferation by inhibiting yes‐associated protein (YAP), a recognized oncogene in glioma, independent of Hippo pathway. ↓orthotopic tumor progression and prolonged survival of tumor‐bearing mice. ↑inhibitory effect on glioma growth both in vitro and in vivo, suggesting the synergism of both agents.	[28]
Kaina et al.	Methadone	The human GB cell lines LN‐229 and A172	the data show that methadone on GB cells in vitro is cytotoxic and induces apoptosis/necrosis at doses that are above the level that can be achieved in vivo. It is not genotoxic, and does not ameliorate the cell killing or the senescence‐inducing effect of TMZ (no synergistic effect), indicating it has no impact on TMZ‐induced signaling pathways.	[29]
Honc et al.	Methadone	Cell lines C6 (rat GB) and T98G and U251 (human GB) cell lines	potentiates the effect of TMZ on rat C6 GB cells and on human U251 and T98G GB cells ↑cell mortality by approximately 50% via a mechanism of action independent of opioid receptors. affecting mitochondrial potential, the level of oxidative stress, intracellular Ca^2+^ concentration and possibly intracellular ATP levels.	[30]
Oppermann et al.	Methadone	Cell culture model of primary GB and fibroblast cell cultures derived from GB patients.	D,L‐methadone reduces GB cell viability only when concentrations are used that had been reported to be toxic to patients and as there were no interactions observable combining it with standard therapy, a recommendation for the use of D,L‐methadone in GB therapy cannot be given	[31]
Latzer et al.	Methadone	Glioma cell lines (LN‐229, A172, U87 and LN‐308) as well as GB initiating cells (GIC; S24, T325, T1, L2 and T269)	Methadone alone has a poor effect on glioma cells at extremely high concentration. The addition of methadone to TMZ does not provide further anti‐tumoral effect compared to TMZ alone and the effect on GIC may even be adverse. Receptor expression on patient tissue is a rare exemption	[32]
Lorio et al.	Morphine	A total of 136 Foxn1 nude mice	GB cells growth is not influenced by morphine treatment and this study show that morphine is an inhibitor of the activity of P‐gp efflux transporter who is markedly expressed on BBB. the combination of morphine with lower dose of TMZ result in a cytostatic effect on tumor growth over the period of the pharmacological treatments.	[33]
Kumari et al.	Nitroxoline	TMZ‐resistant cell lines in vitro	↓expression of APE‐1 after NTX treatment which trigger apoptosis in TMZ‐resistant cells. ↓the colony forming capacity and migration capacity of TMZ‐resistant cells. ↓the tumor growth in TMZ‐resistant GB mice. ↑apoptosis through the decreased expression of APE‐1 in TMZ‐resistant GB in vitro.	[34]
Cho et al.	Nitroxoline	The human GB cell lines LN229 and U87MG	recovery of TMZ‐induced morphological changes, a reduced number of colonies and a decreased rate of migration capacity in TMZ‐resistant cells after NTX treatment. ↓The expression of APE‐1 in TMZ‐resistant cells after NTX treatment compared with those without treatment. ↓tumor growth in TMZ‐resistant GB mice. ↑ADC in the NTX‐treated TMZ‐resistant GB mice compared to the control group.	[35]
He et al.	Tamoxifen	The human glioma cell lines U87‐MG, U251, and A172	direct inhibition of mitochondrial complex I might underlie the proapoptoitc action of tamoxifen in glioma cells	[36]
Hassan et al.	Azithromycin	Human GB cells U87	The cytotoxicity was potentiated when the cells were cotreated with TMZ and AZI compared to TMZ alone, but was markedly lower than AZI alone. TMZ + AZI exerted a competitive inhibition of cell growth and TMZ partially repressed the cytotoxicity of AZI. These findings suggest that simultaneous treatment may not be beneficial, but alternative approaches, such as pretreating with AZI before TMZ, could be worth exploring.	[37]
Zandi et al.	Ciprofloxacin	GB A‐172 cell line	↓mitochondrial topoisomerase II thus affecting cellular energy metabolism. ↓the proliferation of Jurkat cells. CPF exhibited cytotoxic properties and enhanced the inhibitory effects of TMZ on the cell viability of human GBA‐172 cells.	[38]
Eda et al.	Clindamycin	Human GB cell lines (U251, T98G, and LN229)	CLD inhibited the mTOR signaling, which is frequently dysregulated in cancers. Moreover, CLD enhanced the susceptibility of GB cells toward TMZ‐based therapy, which is presently a standard treatment for GB. This study speculate that a CLD and TMZ combination therapy prevented the development of drug resistance by reducing MGMT through mTOR signal downregulation.	[39]
Drljača et al.	Diazepam	U87 human GB	The results showed that DIA antagonized TMZ's anti‐tumor effects, significantly reducing TMZ‐induced apoptosis and enhancing mitochondrial respiration and bioenergetic activity in GBM cells. These findings suggest that concurrent use of DIA may diminish TMZ efficacy, although DIA's independent effects on cell cycle arrest and tumor metabolism raise the possibility of exploring its role as a maintenance therapy after completion of TMZ treatment.	[40]
Ma et al.	Fluoxetine	Glioma cell lines	FLT inhibited the proliferation of various glioma cell lines, and rat glioma C6 cells had a highly sensitive response to the addition of FLT. ↑the early apoptosis rate, induced typical apoptotic morphology in the C6 cells and activated caspase‐3 with no change in the mitochondrial membrane potential. activated the ERS marker, CHOP.	[41]
Song et al.	Fluoxetine	Glioma cells	FLT attenuated MGMT expression by disrupting NF‐κB signaling and sensitized glioma cells to TMZ, which may warrant further investigation toward possible clinical application in MGMT‐expressing glioma	[42]
Lun et al.	Disulfiram	BTIC lines/Six‐ to 8‐week‐old female SCID mice (CB17)	The combination of DSF‐Cu markedly enhanced the antitumor efficacy of TMZ in vitro and, notably, extended in vivo survival in patient‐derived BTIC models. DSF‐Cu exerts a functional inhibition on DNA repair pathways, thereby potentiating the cytotoxic effects of DNA‐alkylating agents and radiation therapy. Moreover, DSF‐Cu suppresses proteasome activity, further amplifying the therapeutic efficacy of DNA‐damaging agents.	[43]
Triscott et al.	Disulfiram	GB SF188 (TMZ resistant), adult GB U251 (partial TMZ sensitivity), and normal human astrocyte cells GB4 and BT74 GB cells	DSF suppressed growth and self‐renewal of primary cells from 2 GB tumors. DSF inhibited the expression of PLK1 in GB cells. Likewise, PLK1 inhibition with siRNA, or small molecules (BI‐2536 or BI‐6727) blocked growth of TMZ resistant cells.	[44]
Bota et al.	Bortezomib	Glioma cell lines (U21 and D54)	bortezomib activity was directly proportional with the cells' baseline proteasome activity. Combined administration of bortezomib and bevacizumab in athymic mice bearing subcutaneous malignant glioma xenografts produced a more pronounced inhibition of tumor growth and a greater extension of survival compared to treatment with either agent alone.	[45]
Tang et al.	Bortezomib	Human GB‐derived U87, U251, LN229, A172, SF295, and astrocytoma‐derived SF268 cell lines	bortezomib showed synergistic effect with TMZ in vitro and sensitized glioma to TMZ treatment both in vitro and in vivo. bortezomib reduced both the mRNA and protein levels of Forkhead Box M1 (*FOXM1*) and its target gene *Survivin*.	[46]
Vlachostergios et al.	Bortezomib	The human GB cell lines T98G and U87	proteasome inhibition by bortezomib overcomes MGMT‐mediated GB chemoresistance, with scheduling of administration being critical for obtaining the maximal tumoricidal effect of combination with TMZ. This study revealed a new role for bortezomib as a potent MGMT inhibitor in GB through interference with NFκB, MAPK, STAT3 and HIF‐1α pathways.	[47]
Rahman et al.	Bortezomib	P3 patient‐derived GB cells, as well as the tumor cell lines U87, HF66, A172, and T98G	bortezomib treatment abrogated autophagic flux indicated by the accumulation of LC3A/B‐II and p62 (SQSTM1) positive autophagosomes that did not fuse with lysosomes and thus reduced the degradation of long‐lived proteins. bortezomib synergistically enhanced TMZ efficacy by attenuating cell proliferation, increased DNA double‐strand breaks, and apoptosis in an autophagy‐dependent manner.	[48]
Kim et al.	Penfluridol	Five GSCs CSC2, X01, 0315, 528NS, 83NS Five‐ to six‐week‐old female Balb/c nude mice	PFD treatment reduced cancer cell migration and invasion by reducing the Integrin α6 and uPAR levels and suppression of the expression of EMT factors, vimentin and Zeb1. PFD with TMZ significantly suppressed tumor growth and prolonged survival in vivo.	[49]
Dong et al.	Everolimus	Human glioma cell lines	Everolimus significantly inhibits the proliferation of glioma cells and promotes the occurrence of autophagy. Combined use of TMZ and everolimus significantly enhances the sensitivity of TMZ to glioma cells, inhibits cell proliferation, and promotes autophagy better than TMZ alone	[50]
Arriaga et al.	Minocycline	GB (U87) and human umbilical vein endothelial cells (HUVEC)	combination of MINO and TMZ resulted in additive effects compared to each drug alone and thus, the dual delivery of both drugs from a local delivery system may be a promising therapy for GB	[51]
Bow et al.	Minocycline	Fischer 344 rats	These results show that MINO delivered locally potentiates the effects of both radiotherapy and oral TMZ in increasing median survival in a rodent glioma model.	[52]
Ivanova et al.	Lomitapide	The immortalized human GB cell line U251, immortalized NHAs, and the human embryonic kidney cell line HEK293	Treatment of TMZ‐resistant glioma cells with the lipid‐lowering drug, lomitapide, resulted in CoQ10 depletion and cellular ROS accumulation, which primes glioma cells for ferroptotic death	[53]
Barrette et al.	Verteporfin	Up to 8 low‐passage patient‐derived GB cell lines with distinct genomic drivers	Combined anti‐invasive and anti‐proliferative efficacy for VP with survival benefit in preclinical GB models, indicating potential therapeutic value of this already FDA‐approved drug if repurposed for GB patients	[54]
Casati et al.	Verteporfin	GB cell lines (U87MG, T98G and A172)	Three GB cell lines (U87MG, T98G and A172) are characterized by high levels of autophagy and the inhibition of the Hippo pathway with VP and especially the combination DOX‐VP reduced the activation of this protumoral molecular mechanism in GB cell lines.	[55]

## 2 Antimicrobial Drugs

### 2.1 Chloroquine

Chloroquine (CQ), which is used for autoimmune and viral diseases, including the treatment of malaria, has a high ability to cross the blood–brain barrier (BBB) and was approved by the FDA in 1949 [56]. CQ, in combination with TMZ, leads to increased formation of autophagic vacuoles and enhances its efficacy against GB. Still, inhibition of autophagy at an early stage, achieved by targeting Beclin 1 or pretreatment with 3‐methyladenine, neutralizes the enhanced effect of the combination treatment. This combination treatment modulated the apoptotic pathway by elevating p53 levels. In contrast, in U373 cells overexpressing mutant p53, despite TMZ‐induced autophagy and CQ‐mediated accumulation of autophagic vacuoles, neither proliferation inhibition nor apoptosis induction increased within 3 days. However, long‐term treatment (9–10 days) effectively reduced clonal and cell growth and induced G2‐M cell cycle arrest, effects that were also abolished by Beclin 1 inhibition [5]. Furthermore, a study by Hori et al. examined the effects of CQ and TMZ on mitochondrial reactive oxygen species (ROS) and cell death using MitoSOX red fluorescence in rat C6 glioma cells, showing that TMZ alone (100–1000 μM) did not affect mitochondrial ROS levels and did not induce cell death in C6 cells, but co‐treatment with CQ (10 μM) significantly increased mitochondrial ROS and cell death. Addition of antioxidants significantly reduced CQ‐induced cell death in TMZ‐treated cells, suggesting a role for mitochondrial ROS in this process [6]. TMZ reduces MitoTracker Green fluorescence and mitochondrial protein levels in cells, and CQ prevents this TMZ‐induced damage [6]. Furthermore, the combination of TMZ and CQ resulted in enhanced co‐localization of EGFP‐LC3 dots with mitochondria [6]. CQ also enhanced TMZ‐induced cytotoxicity by increasing cellular ROS and inhibiting mitochondrial autophagy in U87‐MG cells [6].

In a study by Compter et al. patients received daily oral CQ starting 1 week before chemotherapy (TMZ 75 mg/m2/day) and continuing until completion of radiotherapy (59.4 Gy in 33 sessions). Thirteen enrolled patients received CQ doses of 200 mg (*n* = 6), 300 mg (*n* = 3), or 400 mg (*n* = 4). A total of 44 adverse events related to CQ were recorded, some of which led to TMZ discontinuation or adjuvant cycle delays, including electrocardiogram QTc prolongation, irreversible blurred vision, and nausea/vomiting. The median overall survival (OS) was 16 months (range: 2–32 months), with a median survival of 11.5 months in EGFRvIII‐negative patients and 20 months in EGFRvIII‐positive patients. This study concluded that 200 mg of CQ daily is the maximum tolerated dose when combined with concurrent radiotherapy and TMZ in newly diagnosed GB. Despite the known side effects of CQ, the combination of TMZ and radiotherapy at doses commonly used for rheumatoid arthritis may still result in significant adverse effects [57] (Table 2).

**TABLE 2:** Clinical evidence for repurposed drugs combined with temozolomide‐based therapy in patients with glioblastoma.

Author	Drug	Type of study	Effect	References
Compter et al.	Chloroquine	Single‐center, open‐label, dose‐finding phase I trial	CQ's well‐known toxicity profile, it may still elicit significant adverse effects in combination with TMZ and radiotherapy in doses commonly described for rheumatoid arthritis. It is anticipated that more potent and less toxic autophagy inhibitors such as Lys05 or VP will become clinically available throughout the years to come, but these are currently still under investigation in preclinical models.	[57]
Rosenfeld et al.	Hydroxychloroquine	3 + 3 phase I trial design followed by a noncomparative phase II study	A definitive test of the role of autophagy inhibition in the adjuvant setting for glioma patients awaits the development of lower‐toxicity compounds that can achieve more consistent inhibition of autophagy than HCQ.	[58]
Sotelo et al.	Chloroquine	Randomized, double‐blind, placebo‐controlled trial.	CQ may improve mid‐term survival when given in addition to conventional therapy for GB. These results suggest that larger, more definitive studies of CQ as adjuvant therapy for GB are warranted.	[59]
Weller et al.	Valproic acid	Trial, 587 patients	VPA may be preferred over an EIAED in patients with GB who require an AED during TMZ‐based chemoradiotherapy.	[60]
Redjal et al.	Valproic acid	Patients with intracranial gliomas (WHO grade II–IV) who were treated at the Massachusetts General Hospital (MGH) with surgery and TMZ between January 1997 and June 2013	VPA was associated with improved survival in GB in a dose‐dependent manner. However, in grade II and III gliomas, VPA was linked to histological progression and decrease in progression‐free survival	[61]
Watanabe et al.	Valproic acid	112 patients with high‐grade glioma	VPA use during radiotherapy for glioma is associated with delayed hair loss and improvement in survival. Hair loss prevention benefits patients suffering from the deleterious effects of radiation	[62]
Lu et al.	Valproic acid	A systematic review and meta‐analysis (7 electronic databases from inception to April 2018)	Although the pooled evidence currently suggests that VPA may confers a statistically significant OS benefit in GB patients, this may be overestimated by earlier studies and studies with younger cohorts, and influenced by publication bias and study design.	[63]
Kuo et al.	Valproic acid	(cohort study) A total of 2379 patients	VPA at over 84 DDD improved OS in HGGs	[64]
Roh et al.	Levetiracetam	From 2004 to 2016, 322 patients	LEV use was associated with prolonged survival in patients with GB treated with concurrent TMZ chemoradiotherapy	[65]
Bianconi et al.	Levetiracetam + Lacosamide	Observational retrospective single‐cohort study	In patients diagnosed with GB IDH‐WT undergoing chemoradiation therapy, the use of LEV or lacosamide for controlling BTRE does not seem to modify survival. Lacosamide users exhibited a higher IRR of postoperative seizures compared to LEV users, and MGMT promoter methylation appears to be a protective factor.	[66]
Sun et al.	Levetiracetam	Double‐blind and randomized clinical trial conducted in a single center	It is expected that the results of this trial will provide high‐level evidence regarding the clinical benefits of LEV and TMZ combined in the treatment of GB.	[67]
Yoon et al.	Metformin	A randomized, prospective, multicenter, double‐blind, controlled, phase 2 trial (92 patients)	Although the metformin plus TMZ regimen was well tolerated, it did not confer a clinical benefit in patients with recurrent or refractory GB.	[68]
Ohno et al.	Metformin	Multicenter single‐arm phase I/II study conducted in Japan	Results show that the 2250 mg/day metformin appeared to be well tolerated with acceptable toxicity.	[69]
Shenouda et al.	Metformin	Phase II (From April 2015 to November 2020, 50 patients participated in the M‐HART trial and matched with 50 PSMC cohort treated during the same period.)	These results add to growing evidence for the use of Metformin as an adjunct to HART and TMZ especially in M‐MGMT GB.	[70]
Altwairgi et al.	Atorvastatin	Prospective, open‐label, single‐arm, phase II study	Patients received atorvastatin for a median duration of 6.2 (0.3–28) months. At a median follow‐up of 19 months, the PFS‐6 rate was 66%, with a median PFS of 7.6 (5.7–9.4) months.	[71]
Pace et al.	Chlorpromazine	Multicenter phase II single‐arm clinical trial.	The addition of CPZ to standard TMZ in the first‐line treatment of GB patients with unmethylated *MGMT* gene promoter was safe and led to a longer PFS than expected in this population of patients. These findings provide proof‐of‐concept for the potential of adding CPZ to standard TMZ treatment in GB patients with unmethylated *MGMT* gene promoter	[72]
Onken et al.	Methadone	Retrospective study (in 27 patients)	D,L‐methadone can be safely combined with standard glioma chemotherapy without increasing the risk of toxicity or vegetative symptoms such as tachycardia, sweating and restlessness. PFS‐6 in patients with primary GB treated this way seems to be at least comparable to that of historic controls	[73]
Mckean et al.	Minocycline	A phase 1 trial	MINO at 150 mg twice per day is well tolerated with standard chemoradiation in patients with newly diagnosed high‐grade gliomas. PFS was not significantly increased with the addition of MINO when compared to historical controls. NF‐κB activation correlates with microglia levels in high‐grade glioma	[74]
Patel et al.	Tamoxifen	Phase I Clinical Trial (A total of 17 consecutive patients in four cohorts with World Health Organization Grade 3 (*n* = 2) and 4 (*n* = 15) gliomas were given tamoxifen twice daily during 6 weeks of concurrent RT and TMZ.)	MTD of tamoxifen was 100 mg/m^2^ when given concurrently with TMZ 75 mg/m^2^ and RT. Tamoxifen might have a role in the initial treatment of high‐grade gliomas and should be studied in future Phase II trials building on the newly established platform of concurrent chemoradiotherapy	[75]
de Assis et al.	Metformin	Ten studies were included, comprising 3623 patients (systematic review and meta‐analysis)	Patients who received TMZ + MET had a significantly higher OS than patients who received TMZ alone.	[76]

Rosenfeld et al. conducted a 3 + 3 phase I trial followed by a noncomparative phase II study in GB patients after primary resection. Patients received oral hydroxychloroquine (HCQ) at 200–800 mg daily, in combination with radiotherapy and concomitant TMZ, as well as adjuvant therapy. At the highest dose of 800 mg/day, all three patients experienced grade 3–4 neutropenia and thrombocytopenia, and one patient developed sepsis. 600 mg/day of HCQ was determined as the maximum tolerated dose in this regimen. In the phase II group (*n* = 76), the median overall survival was 15.6 months, and the 12‐, 18‐, and 24‐month survival rates were 70%, 36%, and 25%, respectively. Although autophagy inhibition was not consistently achieved at the 600 mg/day dose and no significant improvement in overall survival was observed, this study demonstrated that HCQ inhibited autophagy by increasing the autophagy markers AV and LC3‐II in peripheral blood mononuclear cells [58].

In a study by Sotelo et al. 30 patients with gliomas confined to one hemisphere of the brain, Karnofsky performance score > 70, no comorbidities, and age < 60 years were enrolled. Patients received either oral CQ 150 mg/day for 12 months, starting on day 5 after surgery, or a placebo in addition to standard chemotherapy and radiation therapy. The median survival after surgery was 24 months in the chloroquine‐treated group, compared with 11 months in the control group. At the end of the follow‐up period, six patients in the CQ group were alive for 59, 45, 30, 27, 27, and 20 months, respectively, while three patients in the control group were alive for 32, 25, and 22 months, respectively. Although this difference was not statistically significant, the mortality rate over time was approximately half in the CQ group compared with the placebo group (hazard ratio: 0.52; 95% CI: 0.21–1.26; *p* = 0.139). These findings suggest that CQ may improve mid‐term survival when added to conventional therapy for GB [59] (Table 2). Thus, CQ can reach the central nervous system and has been evaluated with TMZ‐based chemoradiotherapy in early clinical studies; however, its clinical use is limited by dose‐related toxicity. Although preclinical and early clinical findings support further investigation, a definitive survival benefit has not yet been established.

## 3 CNS Drugs

### 3.1 Valproic Acid

Valproic acid (VPA) is a drug used to treat epilepsy and is also used for bipolar disorder and migraine prevention. Active clearance of valproic acid across the blood–brain barrier appears to be the limiting factor for its entry into the brain [77]. Previous experimental studies have shown that the antitumor effects of VPA can be enhanced when combined with other agents [78, 79]. U87 and U251 cells were independently sensitive to VPA or TMZ, and no additional antitumor effect was observed with the combination treatment. However, the antitumor effect of this combination was evident in TMZ‐resistant T98 and U138 cells, indicating that VPA sensitizes TMZ‐resistant GB cells to TMZ. In T98 and U138 cells, VPA led to a dose‐dependent decrease in MGMT levels by inhibiting HDACs, which promote chromatin remodeling [7]. It has been shown that downregulation of MGMT may reduce the repair of alkylating lesions at the O6 position of guanine, thereby increasing TMZ sensitivity in VPA‐treated TMZ‐resistant cells [7].

Another study by Chen et al. showed that, compared with TMZ or VPA alone, co‐treatment with both drugs synergistically induced apoptosis in U87MG cells by increasing p53 and Bax expression, loss of mitochondrial membrane potential, ROS production, and glutathione depletion by decreasing nuclear translocation of nuclear factor‐erythroid p45‐related factor 2, and downregulating the expression of heme oxygenase‐1 and γ‐glutamylcysteine synthetase, compared with TMZ or VPA alone. Pretreatment with N‐acetylcysteine partially reversed the apoptotic effects of the TMZ/VPA combination. Notably, a similar degree of synergy was observed in p53‐mutated Hs683 cells, suggesting that p53 is not the primary determinant of the enhanced proapoptotic effect of the TMZ/VPA combination [8].

In another study by Tsai et al. the molecular mechanisms of TMZ combined with VPA were further investigated using wild‐type and mutant p53 human glioblastoma cell lines. Experiments showed that VPA enhanced the antitumor effect of TMZ by increasing p53 activation and increasing the expression of its downstream proapoptotic protein, PUMA. However, analysis of clinical data showed that the survival of glioblastoma patients treated with TMZ and VPA combination therapy was dependent on p53 status. These findings suggest that glioblastoma patients with wild‐type p53 may specifically benefit from a combination of TMZ and VPA [9]. However, Chen et al. found that VPA increases secretion of amphiregulin (AR), which activates EGFR signaling and promotes TMZ resistance, while AR knockdown or neutralization enhances TMZ sensitivity [10]. The differing results from Tsai et al. and Chen et al. show that VPA can act as both a helper and a hindrance in glioblastoma treatment. As an epigenetic drug, VPA broadly changes gene expression, enhancing p53‐mediated cell death in some tumors while simultaneously activating EGFR survival signaling in others. Ultimately, whether VPA improves or reduces temozolomide effectiveness depends on the tumor's specific genetic profile, underscoring the need for personalized therapy.

In a study by Weller et al. 175 patients (30.5%) were not receiving any antiepileptic drugs (AEDs) at baseline, 277 patients (48.3%) were taking enzyme‐inducing antiepileptic drugs (EIAEDs), and 135 patients (23.4%) were taking non‐EIAEDs. Patients receiving VPA experienced a higher rate of grade 3/4 thrombocytopenia and leukopenia compared with patients not taking AEDs or taking EIAEDs. Overall survival (OS) was similar between patients who initially received any AED and those who did not. However, patients treated with VPA alone (*n* = 97, 16.9%) appeared to benefit more from TMZ plus radiotherapy (hazard ratio [HR] 0.39; 95% confidence interval [CI] 0.24–0.63) in terms of survival compared with patients treated with EIAEDs alone (*n* = 252, 44%; HR 0.69, 95% CI 0.53–0.90) or patients who did not receive AEDs (HR 0.67, 95% CI 0.49–0.93). This study concluded that VPA may be preferable to EIAEDs for GB patients who require AED therapy during TMZ‐based chemotherapy [60]. Moreover, a retrospective study of 359 patients treated with temozolomide, VPA use in glioblastoma (grade IV) was associated with significantly improved overall and progression‐free survival in a dose‐dependent manner. In contrast, in grade II and III gliomas, VPA was linked to a higher risk of progression and possible histological worsening, suggesting that its benefits may be limited to high‐grade tumors and highlighting the need for prospective studies to clarify its role [61]. In a retrospective study by Watanabe et al. on 112 high‐grade glioma patients, those receiving VPA during radiotherapy exhibited significantly delayed onset of radiation‐induced hair loss and markedly improved median overall survival (42.2 vs. 20.3 months) compared to non‐VPA users. These findings suggest that VPA may serve a dual therapeutic role in glioblastoma management, both mitigating the adverse effects of radiotherapy and improving patient survival outcomes, warranting further prospective investigation [62].

Lu et al. conducted a review of available studies, and 7 retrospective cohort studies were eligible for inclusion in the analysis, including 2181 patients with GB at the time of initial diagnosis, of whom 534 (24%) received VPA as part of their treatment. Overall, VPA was associated with a statistically significant improvement in overall survival, meaning an increase in overall survival of 2.4 months (95% CI 1.51–3.21; *p* < 0.01) compared with the control group (hazard ratio [HR] 0.71; 95% confidence interval [CI] 0.56–0.91; *p* < 0.01). Thus, although VPA is generally associated with improved survival in patients with GB, the magnitude of this effect varies and depends on study characteristics and patient populations. The survival benefit was more pronounced in older studies and in younger patients, whereas it was reduced in more recent studies and in older populations; therefore, generalization of these findings to all patients or treatment conditions requires caution [63]. Additionally, in a study by Kuo et al. a total of 2379 patients with high‐grade glioma (HGG) receiving TMZ were analyzed and stratified into VPA (*n* = 1212, VPA ≥ 84 defined daily doses [DDD]) and non‐VPA (*n* = 1167, VPA < 84 DDD) groups, and followed from 1998 to 2013 or until death. The results showed that the VPA group demonstrated a longer mean overall survival compared with the non‐VPA group (50.3 ± 41.0 vs. 42.0 ± 37.2 months; *p* < 0.001), and the greatest improvement in survival was observed in patients aged 18 to 40 years (70.5 ± 48.7 vs. 55.1 ± 46.0 months; *p* = 0.001) [64]. Therefore, VPA is widely used in patients with glioma for seizure control and has a clinically relevant ability to reach the brain; however, its potential interaction with TMZ may depend on MGMT expression, p53 status, and other tumor‐specific factors. Retrospective studies suggest a possible survival association; however, prospective evidence is insufficient, and toxicity should be monitored when VPA is combined with TMZ‐based treatment.

### 3.2 Levetiracetam

Levetiracetam (LEV) is used to treat partial seizures, myoclonic seizures, and primary generalized tonic–clonic seizures in patients with epilepsy. Anticonvulsant drugs (AEDs) are commonly used to manage seizures in glioma patients, and some AEDs, including levetiracetam, have been reported to modulate MGMT expression. Recent pharmacokinetic data also indicate that the brain‐to‐plasma concentration ratio of levetiracetam is close to 1, suggesting effective penetration of the blood–brain barrier [80]. A study by Bobustuc et al. showed that LEV is the most potent MGMT inhibitor among several anticonvulsant drugs with distinct effects on MGMT regulation. In vitro, LEV reduced both MGMT protein and mRNA levels at concentrations within the therapeutic range for seizure prevention (500–1000 mg twice daily, corresponding to serum levels of 20–40 μg/mL). Chromatin immunoprecipitation analysis showed that LEV allowed p53 to bind more readily and more extensively to the MGMT gene promoter by recruiting a repressor complex consisting of mSin3A and the enzyme HDAC1. However, LEV had no MGMT inhibitory activity when p53, mSin3A, or HDAC1 expression was silenced. LEV also inhibited the proliferation of malignant glioma cells and increased their sensitivity to the single‐agent alkylating agent TMZ [18]. Another study by Scicchitano et al. showed that LEV potentiates the effect of TMZ on the proliferation of glioma cancer stem cells (GCSCs) by reducing MGMT expression, promoting HDAC4 nuclear translocation, and activating apoptosis pathways, while being less effective on parental cancer stem cells (PCSCs) [19]. Furthermore, Ni et al. demonstrated that the combination of IFN‐α, LEV, and TMZ had the most potent antitumor activity in MGMT‐positive glioma cell lines (U138, GSC‐1, U118, and T98 G). The triple combination further improved efficacy compared with TMZ + LEV or TMZ + IFN‐α alone in MGMT‐positive cells. Still, it showed no significant effect in vitro on MGMT‐negative lines (SKMG‐4, U87, U373, and U251). Similar results were also observed in subcutaneous mouse models (U138, GSC‐1 vs. SKMG‐4, U87) and in orthotopic models (GSC‐1) in vivo, showing that the addition of LEV and IFN‐α to TMZ significantly prolonged the survival of mice bearing orthotopic GSC‐1 gliomas. The combination also enhanced MGMT inhibition and apoptosis in TMZ‐treated U138 tumors [20].

In a study by Marutani et al. the effects of single and combined TMZ and LEV treatments on cell proliferation and premature senescence in 2 GB cell lines, MGMT‐negative (A172) or MGMT‐positive (T98), were studied. Both LEV and TMZ dose‐dependently reduced cell proliferation in A172 cells, and senescent‐like phenotypes, as assessed by beta‐galactosidase activity, were induced by both agents, with the combined treatment being more effective than either treatment alone [21].

Another study by Roh et al. examined the effect of LEV on survival in patients with glioblastoma cancer receiving concomitant TMZ chemotherapy. From 2004 to 2016, 322 patients with surgically resected, pathologically confirmed IDH wild‐type glioblastoma cancer who received TMZ‐based chemotherapy were analyzed. Patients were stratified according to LEV use at the time of surgery and at the first follow‐up visit. The median survival time in the LEV‐treated group was 21.1 months versus 17.5 months in the no‐LEV group (*p* = 0.003), while the median progression‐free survival (PFS) was 12.3 and 11.2 months, respectively (*p* = 0.017). Other prognostic factors included age, extent of tumor resection, MGMT promoter methylation status, and Karnofsky performance status (KPS). Multivariate analysis showed that age (hazard ratio [HR] 1.02; *p* < 0.001), postoperative KPS (HR 0.99; *p* = 0.002), complete tumor resection (HR 0.52; *p* < 0.001), MGMT promoter methylation (HR 0.75; *p* < 0.001), and use of LEV (HR 0.72; *p* = 0.011) were significantly associated with overall survival [65]. A retrospective study found no survival difference between LEV and lacosamide use during chemoradiotherapy, though MGMT methylation remained protective [66]. A randomized controlled trial is currently underway to further clarify the clinical benefit of LEV plus TMZ in GB [67]. Although extensive laboratory research and some patient data suggest that LEV enhances temozolomide chemotherapy by disabling the tumor's MGMT defense mechanism, conflicting clinical studies show no distinct survival advantage over other seizure medications. This discrepancy likely arises from complex genetic variations between individual tumors, such as p53 and MGMT status, highlighting the critical need for ongoing randomized trials to determine exactly which patients stand to benefit from this specific drug combination.

## 4 Cardiovascular Drugs

### 4.1 Aspirin

Aspirin (ASA) is used for cardiovascular protection and is widely used for pain relief, anti‐inflammation, and prevention of heart attacks and strokes. It is detected in brain tissues after systemic administration, indicating its ability to cross the blood–brain barrier; however, its concentration in the central nervous system was significantly lower than its plasma levels [81]. Combining ASA with TMZ can enhance TMZ's antitumor activity in GB by modulating multiple cell signaling pathways and molecular mechanisms [11]. Experimental studies have suggested that nonsteroidal anti‐inflammatory drugs, including ASA, may interfere with ERK signaling by disrupting the interaction between RAS and c‐RAF. The RAS/RAF/MEK/ERK pathway regulates cell proliferation, survival, and differentiation and is frequently dysregulated in glioblastoma. In experimental models, inhibition of this pathway has been associated with reduced tumor growth and progression [82, 83, 84]. Specifically, one study showed that ASA inhibited the formation of vascular‐like networks and cell migration both alone and after stimulation with vascular endothelial growth factor (VEGF). Notably, ASA can also be combined with TMZ, bevacizumab (BEV), and sunitinib (SUN) to enhance their efficacy. For example, ASA has the strongest effect in combination with TMZ and BEV, resulting in a moderate reduction in the expression of VEGF, VEGFR‐1, and hypoxia‐inducible factor (HIF)‐1α. At the same time, treatment with TMZ and BEV alone resulted in a significant increase in the expression of VEGF, VEGFR‐1, and HIF‐1α compared with the control group. Combination treatment with TMZ, BEV, SUN, and ASA decreased the expression of RAS/MEK/ERK and PI3K/AKT genes compared with the untreated control group and single‐drug treatments [11].

Furthermore, another study by Ming et al. showed that although short‐term ASA treatment increased GLI1 expression (GLI1, a reliable molecular marker of SHH/GLI1 pathway activity, is essential for GB chemoresistance), it could, in combination with TMZ, inhibit GLI1 activation both in vitro and in vivo. At therapeutic doses with limited cytotoxicity, cells survived for 72 h through activation of DNA damage repair mechanisms, which was accompanied by compensatory upregulation of GLI1; thus, low‐dose ASA was repurposed with minimal side effects to overcome TMZ resistance in glioma and reduce cell survival. This study also showed that the combination treatment exerted greater fold changes in U87 cells than in T98G cells, leading to a decrease in cell survival through TMZ‐induced reduction of p‐p65, further inhibition of total and nuclear GLI1 expression, and increased γH2AX levels, both in vitro and in vivo [12]. Given that NF‐κB is activated by genotoxic and alkylating agents [85, 86], and that the SHH/GLI1 signaling pathway has been identified as a target of NF‐κB that promotes resistance to NF‐κB‐mediated apoptosis in cancer [87]. This may contribute to the increased acquired expression of GLI1 in glioma cells in response to TMZ.

Furthermore, the DNA double‐strand break (DSB)‐responsive ATM cascade has been implicated in NF‐κB activation [88, 89]. In addition, ASA combined with TMZ has shown synergistic antitumor effects in glioblastoma models by modulating molecular pathways involved in tumor progression. This suggests that TMZ‐induced NF‐κB activation stimulates GLI1, which in turn enhances DNA repair via ATM, creating a feedback loop that promotes TMZ resistance. Combination treatment with ASA (NF‐κB inhibitor) disrupts this NF‐κB‐GLI1‐ATM feedback loop and increases chemosensitivity by inhibiting GLI1, impairing DNA repair, and reducing ATM‐mediated NF‐κB activation [11, 12]. Therefore, ASA may influence signaling pathways involved in glioblastoma growth and TMZ resistance; however, evidence for TMZ sensitization remains mainly preclinical. Its limited brain exposure and the risk of bleeding, particularly in perioperative patients or patients receiving corticosteroids and anticoagulants, should be considered. Clinical studies are required before aspirin can be considered a TMZ‐modulating treatment.

### 4.2 Amlodipine

Amlodipine was first approved by the U.S. Food and Drug Administration (FDA) in 1992 for the treatment of hypertension and angina pectoris, and is also commonly used to manage high blood pressure and chest pain. Amlodipine is a calcium channel blocker with poor blood–brain barrier penetration. However, some studies suggest that, in combination with TMZ, it may modulate various cell signaling pathways and molecular processes, thereby improving antitumor efficacy in GB. A study by Zhang et al. showed that amlodipine enhances the cytotoxicity of TMZ against GSCs by reducing MGMT expression through suppression of the Akt/GSK3β/β‐catenin pathway, leading to inhibition of glioma stem cell (GSC) viability as well as induction of apoptosis and cell cycle arrest in vitro [22]. In vivo, amlodipine significantly reduced tumor volume and increased median survival in tumor‐bearing mice. Pharmacological inhibition of GSK‐3β with CHIR‐99021 or β‐catenin overexpression also neutralized amlodipine's downregulation of β‐catenin and MGMT expression, thereby abolishing the reduction in cell viability and the increase in temozolomide (TMZ) cytotoxicity in glioma stem cells (GSCs). Intracranial transplantation models also confirmed that the synergy between amlodipine and TMZ is mediated by downregulation of β‐catenin and MGMT, leading to prolonged survival in tumor‐bearing mice [22]. As a result, amlodipine has shown TMZ‐enhancing activity in glioma stem‐cell and animal models, potentially through modulation of the Akt/GSK3β/β‐catenin/MGMT pathway. Nevertheless, amlodipine has limited blood–brain barrier penetration, and the concentrations required for antitumor activity may not be achievable with standard antihypertensive dosing. Clinical studies are needed to evaluate its pharmacokinetics, safety, and efficacy in glioblastoma.

## 5 Antidiabetic Drugs

### 5.1 Metformin

Metformin, a drug used to treat type 2 diabetes, can induce hypoxia‐like conditions in cells by increasing ROS levels, disrupting mitochondrial membranes, and suppressing their biogenesis, according to gene set enrichment analysis (GSEA) [13]. Recent studies also show that metformin can penetrate the blood–brain barrier and affect neurons and glial cells. However, its exact mode of action in the central nervous system (CNS) is still not fully understood [90]. When combined with TMZ, this drug can affect multiple signaling and molecular pathways, reduce cell viability, proliferation, and migration, increase apoptosis, and impair epithelial‐mesenchymal transition (EMT) in three glioblastoma cell lines, including U87, LNZ308, and LN229, thereby enhancing the anticancer activity of TMZ in glioblastoma cells [13].

For example, in TMZ‐resistant LN229 cells, metformin alone or in combination with TMZ reduced the expression of mitochondrial transcription factor A, Twist, and MGMT [13]. In addition, a study by Valtorta et al. showed that metformin increased the sensitivity of the U251 cell line to TMZ and overcame the resistance of T98G glioblastoma cells to TMZ. Combination treatment modulated apoptosis by increasing the Bax/Bcl‐2 ratio and reducing ROS production, and also reduced the expression of the GSC marker CD90 specifically in T98G cells, without affecting CD133 expression [14]. Another study by Yang et al. showed that metformin inhibited the growth of glioblastoma grafts in vivo by reducing SOX2 expression in TMZ‐resistant glioma cells and the neurosphere‐forming capacity of glioblastoma cells [15].

Furthermore, another study by Yu et al. showed that combination treatment with TMZ and metformin synergistically reduced the formation of secondary gliospheres and the expansion of GSCs, and induced apoptosis in glioma cells and GSCs. In addition, metformin effectively inhibits TMZ‐induced AKT activation, and its co‐treatment with TMZ results in decreased mTOR, eIF4E‐binding protein 1 (4EBP1), and S6K phosphorylation [16]. These studies suggests that metformin may help slow tumor growth by targeting key cancer pathways and stem cells, making it a promising add‐on treatment option that warrants further investigation [76, 91].

In a study by Yoon et al. 81 of 92 screened patients were randomly assigned to two groups: a control group that received placebo plus low‐dose TMZ (*n* = 43) (50 mg/m2 daily) and an experimental group (*n* = 38) that received metformin (1000 mg, 1500 mg, and 2000 mg daily, respectively, in the first, second, and third weeks until disease progression) in combination with low‐dose TMZ. The study concluded that although the combination of metformin and TMZ was well tolerated, it did not provide significant clinical benefit in patients with relapsed or refractory glioblastoma [68]. In addition, a study by Ohno et al. identified metformin as a potential therapeutic option for glioma due to its induction of glioma stem cell differentiation and activation of AMPK‐FOXO3, which inhibits tumor formation. Given the dose‐dependent nature of metformin's anticancer effects, a phase I dose‐escalation trial was conducted to evaluate the safety and determine the recommended phase II dose when combined with maintenance TMZ in patients with newly diagnosed glioma. The results showed that metformin at a dose of 2250 mg/day was well tolerated and associated with acceptable toxicity, leading to advancement to a phase II study to evaluate clinical efficacy [69] (Table 2).

In a phase II trial, the addition of metformin to TMZ in a hypofractionated accelerated radiotherapy (M‐HART) protocol was investigated in patients with glioblastoma. The results showed that this treatment was not only tolerable but also significantly improved median survival time (MST) and progression‐free survival (PFS) compared with the control group, with the greatest effect observed in patients with methylated MGMT and complete tumor resection [70]. Drug repurposing, leveraging existing preclinical and clinical data, can reduce research costs and reduce the need for early‐stage studies. For example, in glioblastoma, some repurposed drugs, such as metformin, have reached clinical evaluation more quickly and have shown cost savings. However, comprehensive cost analyses of glioblastoma are limited, and further studies are needed to determine the cost of repurposing these drugs. Metformin has shown TMZ‐sensitizing effects in several preclinical models, including models of TMZ resistance. However, its brain and intratumoral exposure at clinically tolerated doses remain an important concern. Early clinical studies indicate that metformin‐TMZ combinations may be feasible, but clinical efficacy remains uncertain. Renal function, metabolic status, and the risk of lactic acidosis should be considered in future trials.

## 6 Lipid‐Lowering Drugs

### 6.1 Atorvastatin

Atorvastatin is a drug used to treat high cholesterol and prevent cardiovascular disease, and has been shown to cross the blood–brain barrier in experimental models of the BBB. However, it is not yet clear whether it can reach therapeutically relevant concentrations in the brain after standard systemic administration [92]. In a study by Peng et al. atorvastatin alone dose‐dependently inhibited the growth and survival of several glioblastoma cell lines. It significantly enhanced its efficacy in combination with TMZ in vitro and in glioblastoma transplantation models. This study demonstrated that atorvastatin significantly enhanced the efficacy of TMZ in glioblastoma through prenylation‐dependent inhibition of Ras and its downstream signaling, including ERK, pS6, and eIF4E, and that targeting Ras activity with atorvastatin effectively suppressed MEK and other related signaling pathways [17]. In a prospective, single‐arm phase II study, Altwairgi and colleagues evaluated the efficacy of atorvastatin in combination with standard glioblastoma therapy (radiotherapy and TMZ) in patients with a median age of 52 years, 22% of whom were 60 years or older, and 62% were male. Patients received atorvastatin for a median of 6.2 months, and at a median follow‐up of 19 months, the 6‐month progression‐free survival (PFS‐6) rate was 66%, with a median PFS of 7.6 months. Grade ≥ 3 hematologic adverse events included thrombocytopenia in 7% and neutropenia in 12%, and multivariate analysis showed that higher baseline LDL levels were associated with shorter survival (*p* = 0.046). Although atorvastatin did not significantly improve PFS‐6, high LDL was identified as an independent predictor of adverse outcomes in patients with glioblastoma [71]. Atorvastatin has demonstrated preclinical TMZ‐enhancing effects through inhibition of Ras‐related signaling. However, it remains uncertain whether standard systemic dosing provides therapeutically meaningful concentrations in glioblastoma tissue. Available clinical evidence is limited and does not establish a survival benefit. Potential hepatic toxicity, myopathy, and drug–drug interactions should be considered.

### 6.2 Lomitapide

Lomitapide is used to treat homozygous familial hypercholesterolemia (HoFH) and also to reduce LDL cholesterol levels in these patients. A study by Ivanova and colleagues showed that lomitapide can significantly improve the sensitivity to TMZ‐induced ferroptosis. Treatment of TMZ‐resistant glioma cells with lomitapide alone resulted in decreased intracellular ubiquinone (CoQ10) levels and increased sensitivity to TMZ‐induced ferroptosis, and co‐treatment with lomitapide and TMZ significantly prolonged survival and delayed tumor recurrence in a glioma model compared with TMZ monotherapy. These findings suggest that lomitapide may serve as a promising adjuvant therapeutic agent for TMZ‐resistant glioma [53]. Lomitapide has shown potential to increase TMZ‐induced ferroptosis in TMZ‐resistant glioma models. However, evidence is currently limited to preclinical studies, and its ability to reach glioblastoma tissue remains insufficiently characterized. Its known hepatic toxicity and lipid‐related adverse effects may also limit clinical use. Therefore, further pharmacokinetic and safety studies are necessary before clinical translation.

## 7 Antipsychotic Drugs

### 7.1 Chlorpromazine

Chlorpromazine (CPZ) is one of the approved drugs for schizophrenia and is widely used as an antipsychotic. In an in vitro blood–brain barrier model, chlorpromazine showed significant permeability, confirming its ability to cross the blood–brain barrier [93]. Glioblastoma resistance to therapy is associated with several factors, including the presence of highly tumorigenic glioblastoma stem cells (GSCs), which contribute to tumor maintenance, heterogeneity, and treatment failure [94, 95]. It has also been shown that CPZ can reduce the expression of genes important in glioblastoma stem cells, including OCT3/4, SOX2, NANOG, Nestin, OLIG2, and ALDH1A3, and its combination treatment with TMZ regulates signaling pathways and ultimately improves antitumor outcomes in glioblastoma [96].

In a study, Matteoni et al. observed that the combination of CPZ and TMZ, in addition to effectively suppressing the colonization ability and neurosphere formation, also reduced cell survival. At the mechanistic level, TMZ primarily inhibits DNA replication forks and arrests the cell cycle in the G2/M phase, whereas CPZ, in addition to inducing G2/M arrest, appears to prevent premature apoptosis in damaged cells. Furthermore, evidence suggests that autophagy‐dependent cell death also contributes to CPZ‐induced cytotoxicity in glioblastoma cells. Accordingly, the simultaneous combination of TMZ and CPZ is likely effective in overcoming drug resistance in glioblastoma by activating multiple cell death pathways [23]. Furthermore, another study by Matarrese et al. showed that CPZ enhances the cytotoxic effects of the alkylating agent TMZ by reducing Cx43 expression and disrupting the cell cycle arrest required for DNA repair [24].

In a single‐arm, multicenter phase II trial, the effect of adding chlorpromazine (CPZ) to temozolomide (TMZ) in the adjuvant phase of treatment of patients with newly diagnosed glioblastoma with an unmethylated MGMT promoter was evaluated. Results showed that among 41 enrolled patients, 65.8% remained progression‐free at 6‐month follow‐up, with a median progression‐free survival of 8 months. The median overall survival was also reported to be 15 months. In terms of safety, the incidence of adverse events was limited, and only about 10% of patients required dose reduction or discontinuation of CPZ. Overall, the data suggest that adding CPZ to the standard TMZ regimen in this subgroup with a poor prognosis was associated with acceptable survival and tolerability improvements [72] (Table 2). CPZ can cross the blood–brain barrier and has shown activity against glioblastoma cells and stem‐like cells in preclinical models. Nevertheless, convincing clinical evidence for enhancement of TMZ efficacy is lacking. Its sedative, anticholinergic, cardiovascular, and extrapyramidal adverse effects, as well as possible QT prolongation, should be considered when evaluating this combination.

### 7.2 Penfluridol

Kim and colleagues reported that penfluridol (PFD), an oral antipsychotic drug commonly used in the treatment of schizophrenia, exhibits antitumor activity in various types of cancer cells [49]. It can also cross the blood–brain barrier [97]. Forexample, because the drug readily crosses the blood–brain barrier, it can affect glioma stem cells (GSCs), which are implicated in drug resistance in glioblastoma. In five glioma stem cell lines, PFD dose‐ and time‐dependently inhibited proliferation and, at the IC50 concentration, reduced the size and the ability of neurospheres to re‐form and reduced the expression of stemness markers SOX2 and OCT4. It also inhibited migration, invasion, and the EMT process by downregulating α6 integrin and uPAR and suppressing vimentin and Zeb1, a process accompanied by a decrease in GLI1. In an animal model, the combination of PFD with TMZ significantly reduced tumor growth and increased survival, whereas TMZ alone did not show these molecular effects. These results support the potentiating role of PFD in combination with TMZ in the treatment of glioblastoma [49]. Imipramine and penfluridol have shown antitumor activity in laboratory and animal glioblastoma models; however, evidence for clinically meaningful TMZ sensitization remains limited. The ability to achieve effective brain concentrations and the risk of neuropsychiatric, cardiac, and drug‐interaction adverse effects require careful assessment before clinical evaluation.

## 8 Supplement

### 8.1 Melatonin

Recent studies suggest that melatonin contributes to the integrity and function of the blood–brain barrier and may even facilitate drug passage across the blood–brain barrier through its protective and regulatory effects [98]. Melatonin (MEL) in combination with temozolomide (TMZ) can target multiple signaling and molecular regulatory pathways, thereby enhancing antitumor efficacy. In a study by Bostanci et al. combination treatment with TMZ and MEL significantly reduced the viability of glioblastoma and neuroblastoma cells compared to the control group. Notably, the combination of MEL with half the IC50 of temozolomide significantly increased cancer cell death and even showed a stronger effect than some of the comparison groups. The combination was also shown to increase the expression of genes related to antioxidant enzymes (Sod1 and Sod2) and genes involved in DNA repair (Mlh1, Exo1, and Rad18). At the same time, a significant decrease in the expression of Nfkb1 and Pik3cg was observed, indicating the inhibition of survival and inflammatory pathways. This combination treatment caused cell cycle arrest, increased expression of p53 and pro‐apoptotic proteins such as Bax and caspase‐3, and decreased expression of Bcl‐2, an anti‐apoptotic factor [25]. In another study, Tang et al. showed that co‐treatment of melatonin with temozolomide significantly reduced cell proliferation, migration, and invasion compared with single treatments. This synergy occurs through inhibition of the NF‐κB/COX‐2 signaling axis, in which melatonin blocks NF‐κB activation by preventing IκBα phosphorylation and, subsequently, reduces COX‐2 expression, a pathway normally involved in tumor cell survival and inflammation. Furthermore, combination treatment increased the apoptosis process by activating caspase‐3 [26].

In a study by Gil et al. treatment of glioblastoma cell lines with 3 mM melatonin in vitro resulted in a significant reduction in cell proliferation, migration, and invasion. Also, after 48 h, an increase in early apoptosis was observed, accompanied by cell cycle arrest in the G2/M phase and a decrease in the S‐phase cell population. From a metabolic perspective, intracellular lactate levels increased, and extracellular lactate levels decreased; a change associated with decreased expression of the lactate transporter MCT4, the membrane protein CD147, and key enzymes of the aerobic glycolysis pathway. This metabolic rearrangement was accompanied by increased oxygen consumption and intensified mitochondrial reactive oxygen species (ROS) production, ultimately leading to enhanced cell differentiation. The consequence of these changes was an increase in glioblastoma cells' sensitivity to temozolomide, leading to a significant synergistic effect between MEL and TMZ [27]. Melatonin is able to enter the central nervous system and has shown favorable preclinical activity in combination with TMZ. However, the currently available evidence is largely limited to cell and animal models, often using concentrations that may not directly correspond to standard clinical dosing. Clinical trials are needed to determine whether melatonin can safely improve TMZ efficacy in patients with glioblastoma.

## 9 Opioid Agents

### 9.1 Methadone

Methadone, which is used to treat opioid use disorder, is also used as a long‐acting opioid agonist to reduce craving and withdrawal symptoms. In preclinical models, methadone has been shown to cross the blood–brain barrier, and its brain concentration increases significantly when P‐gp‐mediated efflux is inhibited, suggesting that its access to the CNS is limited but possible under conditions of reduced efflux transport [99]. In a study by Kaina et al. the combination of methadone with temozolomide (TMZ) in glioblastoma cells showed that coadministration of methadone with TMZ did not affect methadone‐induced apoptosis (3 days after treatment). That methadone at a low, nontoxic concentration (5 μg/mL) did not affect TMZ‐induced apoptosis (5 days after treatment). However, at the high toxic concentration of methadone (20 μg/mL), the cytotoxic effects of both drugs were additive. Genotoxic studies using Comet and γH2AX assays showed that methadone alone is not genotoxic and does not alter the ability of TMZ to cause DNA damage. Furthermore, methadone neither induces nor reverses TMZ‐induced senescence and does not affect MGMT promoter methylation [29]. In a study by Honc et al. it was found that the potentiating effect of methadone on the efficacy of TMZ in C6 glioblastoma cells was independent of opioid receptor or TLR activity, suggesting that alternative mechanisms are involved. Data suggest that methadone primarily targets mitochondrial function, altering mitochondrial status by modulating intracellular Ca^2+^ levels, increasing oxidative stress, and altering ATP concentration. These changes may contribute to decreased DNA integrity, PARP‐1 degradation, and phosphatidylserine release from the cell membrane. Given the critical role of PARP‐1 in DNA repair and ADP regulation, its disruption could dramatically affect cell survival [30].

Opperman et al. showed that d,l‐methadone at 1 μM reduced fibroblast viability, whereas a higher concentration of about 10 μM was required to have a similar effect on glioma cells. Furthermore, d,l‐methadone did not enhance the antitumor effects of X‐ray radiotherapy, TMZ, or their combination. Given that the effective concentrations in glioma cells were higher than those reported in patients, with tolerability and nontoxicity observed and no synergy with standard therapy, this study concluded that the use of d,l‐methadone is not recommended as a treatment option for glioma [31]. Expression of the μ‐opioid receptor in human glioma tissues and related cell lines is weak, with only a few cells expressing it. Most glioma cell lines and glioma‐initiating cells (GICs) have a methylated MGMT promoter. In a study by Latzer et al. LN‐229 cells showed a moderate G2 arrest after 6 days of methadone treatment, whereas methadone at 1 μg/mL significantly increased T325 cell proliferation. The combination of methadone with TMZ also showed no synergistic or potentiating effect on the antitumor activity of TMZ [32].

A study showed that D, L‐methadone can increase the chemosensitivity of glioma tumor cells. In this trial, the safety and tolerability of adding D, L‐methadone to standard chemotherapy were evaluated in 27 patients, and the dose, treatment duration, and adverse events were recorded. Thirteen patients experienced grade 1–3 nausea at the start of treatment, while four reported ongoing adverse events, including nausea (CTC grade 2, *n* = 1) and constipation (CTC grade 2–3, *n* = 3). The 6‐month progression‐free survival (PFS‐6) rate was 80% in patients with an unmethylated MGMT promoter and 100% in patients with a methylated MGMT promoter [73] (Table 2). Overall, the evidence for methadone as a TMZ‐sensitizing agent remains conflicting. Although enhanced TMZ cytotoxicity has been reported in some cell models, other studies found no additional antitumor effect or observed effects only at concentrations unlikely to be safely achieved in patients; therefore, methadone cannot currently be recommended as a TMZ‐sensitizing strategy.

### 9.2 Morphine

Preclinical data have shown that morphine, which is used to control moderate to severe pain, acts as an inhibitor of P‐glycoprotein (P‐gp). This ATP‐dependent transporter limits the penetration of chemotherapeutic drugs into brain tissue. Inhibition of this transporter can increase the access of TMZ to tumor tissue. In experimental models, morphine alone did not increase tumor growth compared with the control group. Metronomic treatment with TMZ (1.77 mg/kg), either alone or in combination with morphine, inhibited tumor growth from the early stages of treatment. However, the combination of TMZ with morphine significantly enhanced the overall tumor regression process; at day 42, the bioluminescence signal intensity in the group receiving the combination treatment was approximately 2.5‐fold lower than that in the TMZ alone group. Importantly, this improvement in antitumor response was not accompanied by increased systemic toxicity, as body weight changes remained similar across the treatment groups [33].

## 10 Antibiotics

### 10.1 Nitroxoline

Nitroxoline (NTX) is an antibiotic that is highly effective against biofilm infections. The effects of NTX in temozolomide‐resistant glioblastoma models showed that NTX modulated TMZ‐induced morphological changes, reduced colonization ability, and inhibited the migratory capacity of resistant cells. This treatment was associated with a significant reduction in APE‐1 expression, a molecule that plays a key role in DNA repair and drug resistance, compared with untreated cells. In animal models, NTX also inhibited tumor growth. Interestingly, the increase in apparent diffusion coefficient (ADC) values on imaging occurred before the observable reduction in tumor volume. Histological studies showed that reductions in cell density and APE‐1 expression were inversely correlated with increases in ADC, and these changes were associated with improved survival in the NTX‐treated group [35]. Overall, the results suggest that NTX exerts its therapeutic effects by reducing APE‐1, which could be used as a treatment option for TMZ‐resistant glioblastoma (Figure 1). Nitroxoline has demonstrated activity against TMZ‐resistant glioma models, potentially through APE‐1 downregulation. However, evidence remains preclinical, and adequate brain and tumor penetration has not been established. Further pharmacokinetic, safety, and in vivo validation studies are required before considering clinical application in glioblastoma.

### 10.2 Azithromycin

Studies show that azithromycin, used to treat respiratory, skin, and sexually transmitted infections, has produced neuroprotective effects after systemic administration in focal cerebral ischemia models, indicating that this drug reaches the brain tissue and acts within the neural environment [100]. In a study on the U87 cell line, it was also found that azithromycin alone can inhibit cell proliferation and induce apoptosis. Although the combined treatment of temozolomide (TMZ) and azithromycin at concentrations of 0.5 to 225 μg/mL for 48 h caused more cytotoxicity than TMZ alone, the severity of this effect was still less than that of azithromycin monotherapy. Finally, it was shown that the presence of TMZ reduces the severity of azithromycin‐induced toxicity, suggesting antagonistic effects between the two drugs in U87 cells [37] (Figure 1). Therefore, evidence supporting azithromycin as a TMZ sensitizer is limited and uncertain. In the available study, the TMZ–azithromycin combination did not clearly outperform azithromycin alone. Potential cardiac toxicity, including QT prolongation, and drug–drug interactions should also be considered. Additional studies are needed before this combination can be proposed for glioblastoma treatment.

### 10.3 Ciprofloxacin

Fluoroquinolone antibiotics, including ciprofloxacin, can penetrate the cerebrospinal fluid, and ciprofloxacin (CPF) is used to treat bacterial infections, including urinary tract and respiratory tract infections. In one study, CPF demonstrated cytotoxicity and potentiated the inhibitory effects of TMZ on the viability of human A‐172 GB cells. These findings suggest that CPF has a potent antiproliferative effect in A‐172 cells, both alone and in combination with TMZ [38] (Figure 1), however, these findings are preliminary and require confirmation in clinically relevant animal models.

### 10.4 Minocycline

Minocycline (MINO), which has been used for many years as a tetracycline antibiotic in the treatment of infections such as acne and rosacea, has high blood–brain barrier penetration due to its lipophilic properties and has also attracted attention in recent years for its non‐antibiotic properties [101]. In an in vitro study, the effects of MINO and temozolomide (TMZ) were investigated individually and in combination on U87 cells and HUVEC endothelial cells. The results showed that minocycline can induce apoptosis and autophagy in glioma cells by inhibiting angiogenesis‐related processes and increasing the sensitivity of endothelial cells to treatment. Combined treatment with MINO and TMZ caused a greater decrease in viability in both cell lines than either single treatment alone, indicating an additive effect between the two drugs [51].

In a preclinical study by Bow et al. the effect of minocycline‐containing polymer implants in a rodent glioma model was investigated. Local administration of these implants on days 3 and 5 after tumor implantation increased median survival from 14 to 19 days, a statistically significant difference. When MINO polymer was combined with radiotherapy on day 5, median survival increased by 139% compared with radiotherapy alone. Interestingly, there was no significant difference between minocycline administration at the same time as radiotherapy and 2 days before, suggesting that the short time interval surrounding radiotherapy is unlikely to have a decisive impact on outcome. Also, the combination of topical MINO with oral temozolomide increased survival by approximately 38% compared with TMZ alone [52] (Figure 1).

In a clinical study by McKean et al. the safety and efficacy of minocycline were evaluated in patients with newly diagnosed high‐grade glioma who received standard therapy. In this trial, a dose of 150 mg twice daily was determined to be the maximum tolerated dose. Only one case of severe nausea and dizziness was reported, and two patients had platelet reductions that led to temozolomide discontinuation; however, overall, the treatment was considered clinically tolerable. 60% of patients had longer progression‐free survival than predicted, but this did not meet the prespecified efficacy threshold and did not show a significant improvement compared with historical data. Clinical symptoms increased after radiation therapy, but their severity remained mostly mild. Molecular studies also did not show a significant association between biomarkers and PFS, although a correlation was observed between NF‐κB pathway activation (as measured by P‐p65 expression) and the presence of microglia as indicated by IBA‐1 [74] (Table 2). It can be concluded that, although minocycline has been investigated in preclinical models and early clinical settings in combination with TMZ and radiotherapy and it has favorable CNS penetration, the evidence for improved antitumor efficacy remains limited. Its role should therefore be considered investigational until prospective clinical studies establish a clear benefit.

### 10.5 Clindamycin

Clindamycin (CLD), which is used to treat bacterial infections, shows limited penetration of the intact blood–brain barrier, and measurable concentrations in the cerebrospinal fluid are observed mainly under conditions of meningeal inflammation [102]. However, this drug has recently been investigated as a potential treatment option for glioblastoma. In a study by Eda et al. CLD was found to have significant cytotoxic effects on glioblastoma cells, and inhibition of the mTOR pathway, which is active in many tumors, was identified as the main mechanism of this effect. Also, combining CLD with temozolomide (TMZ) increased cell sensitivity to treatment by reducing MGMT expression, an enzyme involved in TMZ resistance. In other words, CLD could increase glioblastoma cells' sensitivity to TMZ by suppressing the mTOR pathway and thereby preventing the development of drug resistance [39] (Figure 1), Nevertheless, its ability to achieve effective glioblastoma‐tissue concentrations and its clinical efficacy remain unknown. Further in vivo and clinical studies are necessary before clinical use can be considered.

## 11 Hormone Therapy

### 11.1 Tamoxifen

Tamoxifen is the mainstay of hormonal therapy in breast cancer. Its antitumor effects are primarily attributed to inhibition of the estrogen receptor (ER) pathway, especially in patients with ER‐positive tumors, and it can cross the blood–brain barrier [103]. Other antitumor effects include increased production of reactive oxygen species, activation of caspases, increased calcium influx, and activation of signaling pathways such as protein kinase C, TGFβ, JNK‐1, and caspase‐3 [104, 105, 106, 107, 108]. A study by He et al. suggested that tamoxifen may mediate its proapoptotic action in glioma cells by directly inhibiting complex I of the mitochondrial respiratory chain [36]. However, in a clinical trial involving 77 GB patients, high‐dose tamoxifen combined with radiation produced survival outcomes similar to standard treatment, but was associated with a lower incidence of thromboembolic events than is typically observed in this population. Although this combination did not represent a major efficacy advance on its own, tamoxifen's favorable safety profile warrants further investigation in combination regimens with other antitumor agents [109]. Moreover, it has been shown that coadministration of tamoxifen and TMZ is well tolerated and may enhance the efficacy of a dose‐dense TMZ regimen as a second‐line treatment strategy [110]. Although the practical relevance of the study by He et al. is limited due to the lack of in vivo human GB xenograft experiments, the findings provide further support for future in vivo research and clinical trials evaluating tamoxifen as a treatment option for GB [36].

In a study by Patel et al. 17 consecutive patients with grade 3 (*n* = 2) and grade 4 gliomas (*n* = 15) were treated with concurrent radiotherapy (RT) and TMZ, with tamoxifen twice daily for 6 weeks. Results were that in group 4, of the six patients who received tamoxifen at a dose of 125 mg/m2, one patient dropped out and another developed grade 4 thrombocytopenia, a dose‐limiting toxicity (DLT) that necessitated the enrollment of three additional patients. Deep vein thrombosis occurred in the sixth patient, setting the maximum tolerated dose (MTD) at 100 mg/m2, which was safely tolerated when combined with TMZ (75 mg/m2) and RT [75], however, evidence of clinical benefit remains limited and inconsistent. The potential for thromboembolic events, endocrine effects, and drug interactions should be considered when assessing tamoxifen‐based combination therapy.

## 12 Benzodiazepine

### 12.1 Diazepam

Diazepam (DIA) was initially approved for the treatment of anxiety and is also widely used for conditions such as seizures, muscle spasms, and alcohol withdrawal. One study showed that free diazepam reaches measurable intermediate concentrations in the brain, indicating penetration of the blood–brain barrier and association with central nervous system effects [111]. DIA has been reported to interact with peripheral benzodiazepine receptors (PBR, also known as the translocator protein, TSPO), which are expressed in the outer mitochondrial membrane and have been implicated in cellular processes beyond their classical role in benzodiazepine binding [112, 113]. Benzodiazepines appear to exert pro‐apoptotic effects in tumor cells through PBR and regulation of mitochondrial membrane potential [113, 114]. Consistent with these findings, a study by Drljača et al. showed that U87 GB cells underwent apoptosis after treatment with TMZ or DIA alone. However, when used together, diazepam actually reduced temozolomide's ability to kill tumor cells by lowering apoptosis and boosting the cells' energy production [40], indicating that DIA should not be considered a TMZ‐sensitizing agent based on the currently available evidence. In addition, sedation, cognitive impairment, respiratory depression, tolerance, and dependence limit its potential as an anticancer adjunct.

## 13 SSRI/Antidepressant

### 13.1 Fluoxetine

Fluoxetine, which is widely used in the treatment of depression, including in cancer patients, has also shown significant antitumor effects in recent studies. A 2023 study showed that fluoxetine crosses the blood–brain barrier in mice and that organic cation transporters (OCT1/3) are involved in its stereoselective uptake into brain tissue [115]. FLT significantly and dose‐dependently inhibited proliferation in several glioma cell lines, including rat C6 cells, which showed high sensitivity. It also led to an early increase in apoptosis and caspase‐3 activation, without affecting mitochondrial membrane potential. Further studies indicate that this drug increases CHOP expression, a key marker of endoplasmic reticulum stress, and is associated with activation of the PERK–eIF2α–ATF4 pathways. Combination treatment with fluoxetine and temozolomide enhanced cytotoxicity in C6 cells, and a synergistic effect between the two agents was observed. Importantly, inhibition of CHOP abolished this synergy, a finding that highlights the pivotal role of CHOP‐dependent pathways in increasing glioma cells' sensitivity to temozolomide. These results indicate that activation of endoplasmic reticulum stress could be an effective mechanism for improving the response to chemotherapy in glioma [41].

In a study by Song et al. it was shown that fluoxetine can significantly reduce MGMT expression in glioma cells independently of GluR1 receptor function. It also attenuated MGMT transcriptional regulation via this pathway by inhibiting NF‐κB/p65 signaling both in vitro and in vivo, and increased the sensitivity of MGMT‐expressing glioma cells to temozolomide, thereby increasing temozolomide‐induced apoptosis and reducing tumorigenicity in vitro. Importantly, inhibition of MGMT or IKKβ neutralized this synergistic effect, indicating that reduced MGMT expression plays a crucial role in sensitizing cells to temozolomide [42], however, clinical evidence in glioblastoma is lacking. Possible interactions with other central nervous system medications, the risk of serotonin‐related adverse effects, and patient‐specific psychiatric considerations should be evaluated in future studies.

### 13.2 Imipramine

In a study, imipramine, which is used to treat depression, was shown to be effluxed by the P‐glycoprotein efflux transporter at the blood–brain barrier. The data showed that inhibition of P‐gp resulted in increased brain accumulation of imipramine, suggesting that the drug can cross the blood–brain barrier, but its access to the brain may be regulated by P‐gp‐dependent transport mechanisms [116]. A study by Wang and colleagues showed that imipramine significantly inhibited the proliferation of immortalized and primary glioma cells by targeting Yes‐associated protein (YAP), a known glioma oncogene independent of the Hippo pathway, and led to increased subcellular translocation from the nucleus to the cytoplasm. Although imipramine treatment significantly reduced orthotopic tumor progression and prolonged survival in tumor‐bearing mice, exogenous YAP overexpression partially reversed imipramine's inhibitory effects on glioma progression. Notably, compared with imipramine or TMZ monotherapy, combination treatment with imipramine and TMZ resulted in enhanced inhibition of glioma growth both in vitro and in vivo, suggesting a synergistic effect of the two agents [28].

## 14 Anti‐Alcoholism Agent

### 14.1 Disulfiram

The Food and Drug Administration approved Disulfiram for the treatment of alcohol use disorder and is currently used as an adjunct to alcohol withdrawal. However, its pharmacological properties, including its ability to cross the blood–brain barrier, relatively low toxicity profile, and affordable cost, have attracted the attention of researchers as a treatment for brain tumors. In primary glioblastomas, MGMT promoter methylation status remains the most reliable biomarker for predicting treatment response [2, 43, 117]. Patients with an unmethylated MGMT promoter typically show limited response to temozolomide, highlighting the need to develop alternative or adjunctive therapeutic strategies. In vitro studies and animal models have shown that DSF‐Cu can enhance the antitumor effects of temozolomide in brain tumor‐initiating cells, regardless of MGMT status. These findings suggest that patients with unmethylated MGMT may benefit from adding DSF‐Cu to their treatment regimen.

Furthermore, the potential of DSF‐Cu to reduce the dose of TMZ while maintaining therapeutic efficacy could reduce drug‐related toxicities and reduce the emergence of mutational phenotypes associated with progression. Given the recently elucidated mechanism of action of DSF‐Cu, particularly its ability to inhibit DNA repair, it would be valuable to investigate its use in both the neoadjuvant and adjuvant settings for newly diagnosed and relapsed patients. By acting as a potential radiosensitizer during radiotherapy, DSF‐Cu could enhance treatment efficacy and improve treatment outcomes [43]. Enhancing the therapeutic activity of TMZ and/or BCNU would be advantageous; however, such combinations necessitate evaluation of the safety and toxicity profile of DSF–Cu in a phase I clinical setting. Furthermore, research indicates that disulfiram reduces the self‐renewal and proliferation capacity of glioblastoma cells, even in temozolomide‐resistant tumors with unmethylated MGMT and high PLK1 expression, by downregulating their expression [44], However, the clinical efficacy of this approach remains unproven.

## 15 Proteasome Inhibitors

### 15.1 Marizomib

Proteasomes are critical regulators of cancer cell physiology, and their inhibition has emerged as a promising therapeutic approach for glioblastoma. Although several proteasome inhibitors (PIs) have been approved for the treatment of other cancers, their efficacy in glioblastoma is limited due to poor penetration of the blood–brain barrier. Marizomib (MZB) is a second‐generation, irreversible proteasome inhibitor that, unlike other proteasome inhibitors, can cross the blood–brain barrier, making it a potential candidate for the treatment of brain tumors. Marizomib (MZB) is a second‐generation, irreversible proteasome inhibitor that, unlike other proteasome inhibitors, can cross the blood–brain barrier, making it a potential candidate for the treatment of brain tumorsThe antitumor effects of marizomib in GBM cell lines LN229 and U118 showed that marizomib significantly reduced cell viability and induced apoptotic cell death by activating caspase‐3 and increasing the expression of cleaved PARP, Noxa, cytochrome c, and DR5 in GBM cells. In addition, the drug induced endoplasmic reticulum (ER) stress by increasing the expression of GRP78, IRE1α, p‐EIF2α, p‐SAPK/JNK, CHOP, ATF6α, and ATF4. In contrast, no increase in ROS production or in the expression of ERO1α, LC3 II, Beclin 1, or ATG5 was observed, indicating that oxidative stress and autophagy were not involved in marizomib‐induced cell death [118]. In a preclinical study, Severin et al. investigated the mechanisms underlying marizomib's action in patient‐derived GBM spheres and evaluated several combination strategies to enhance its antitumor effects. Marizomib dose‐dependently inhibited all three proteolytic subunits of the 20S proteasome, and this inhibition was associated with a decrease in cell proliferation compared with the control group (DMSO). In addition, marizomib treatment induced a transient induction of unfolded protein response (UPR) genes and a sustained increase in SQSTM1 expression. Combination therapy with HDAC inhibitors, such as panobinostat and vorinostat, resulted in synergistic effects on UPR genes and greater suppression of cell proliferation. Marizomib can increase survivin levels, an anti‐apoptotic protein, and its combination with IAP inhibitors such as AZD5582 and LCL161 enhanced the antiproliferative effect compared to marizomib alone [119]. However, these studies showed that marizomib has anticancer effects, and its potential in combination with TMZ warrants further investigation. Moreover, its safety profile and CNS delivery should be carefully evaluated.

### 15.2 Bortezomib

A major translational limitation is the limited penetration of bortezomib into the central nervous system, which may restrict the clinical applicability of otherwise promising preclinical findings. Despite the limited central nervous system (CNS) penetration of bortezomib, which is used to treat multiple myeloma, Botha and colleagues showed that this drug induces caspase‐3 activation and apoptotic cell death in both glioma cell lines and GSCs derived from malignant tumor specimens. Importantly, TMZ‐resistant glioma cells remained sensitive to proteasome inhibition, such that bortezomib activity was directly related to basal proteasome levels. Proteasome inhibition also stimulated HIF‐1α and VEGF production in malignant GSCs, thereby increasing endothelial cell growth [45, 120].

Tang et al. reported that bortezomib, in addition to inhibiting the viability and proliferation of U251 and U87 glioma cells in a dose‐ and time‐dependent manner by inducing apoptosis and cell cycle arrest, suppressed spheroid growth, colony formation, and proliferation of glioma stem cells. Combination treatment with bortezomib and TMZ increased glioma sensitivity to TMZ both in vitro and in vivo. Bortezomib reduced the mRNA and protein levels of Forkhead Box M1 (FOXM1) and its downstream target Survivin. The FOXM1‐Survivin axis was significantly upregulated in TMZ‐insensitive glioma cell lines and in high‐grade glioma (HGG) patients, with FOXM1 and Survivin expression levels positively correlated and associated with poor prognosis [46].

Vlachostergios et al. also showed that the development of drug resistance after standard TMZ chemotherapy in glioblastoma cancer is associated with poor patient prognosis and is mediated in part by MGMT. The 26S proteasome post‐translationally regulates MGMT expression, and proteasome inhibition with bortezomib induces growth arrest and apoptosis in glioblastoma cells. The results showed that optimal cytotoxicity in T98G cells was achieved by administering bortezomib before TMZ. In contrast, the reverse sequence was more effective in U87 cells. Maximal efficacy was associated with reduced MGMT expression, reduced IκBα‐mediated nuclear accumulation of NFκB, and attenuation of p44/42 MAPK, AKT, and STAT3 signaling, as well as stabilization of inactive p53 and HIF‐1α [47]. Moreover, another study found that bortezomib blocks autophagic flux, leading to accumulated damaged proteins, increased DNA damage, and enhanced apoptosis, thereby sensitizing GBM cells to TMZ in an ATG5‐dependent manner. Loss of autophagy (ATG5 knockout) reversed bortezomib's effects, confirming that bortezomib enhances TMZ efficacy by inhibiting protective autophagy [48], however, limited CNS penetration represents a major obstacle to clinical translation.

## 16 mTOR Inhibitors

### 16.1 Everolimus

Dong et al. investigated the effects of the mTOR inhibitor everolimus, used to treat advanced renal cell carcinoma, on glioma cell proliferation, autophagy, and TMZ sensitivity. CCK‐8 assays showed that everolimus inhibited glial cell proliferation in a time‐ and dose‐dependent manner. Western blot analyses revealed a concentration‐dependent increase in autophagy markers, including LC3‐II and the LC3‐II/I ratio, accompanied by a gradual decrease in p62 expression compared with the control group. Treatment with TMZ alone or in combination with everolimus for 48 h inhibited glioma cell proliferation in a dose‐dependent manner, demonstrating that the combination was more effective than TMZ monotherapy. Furthermore, in the TMZ‐everolimus group, Beclin‐1 expression and the LC3‐II/LC3‐I ratio were increased compared with TMZ alone or the control group, while p62 levels were decreased [50]. However, mTOR inhibition may induce autophagy with context‐dependent effects, and preclinical findings may not predict clinical benefit. Limited evidence from glioblastoma trials and potential metabolic, hematologic, and infectious adverse effects warrant cautious clinical evaluation.

## 17 Photodynamic Therapy Agents

### 17.1 Verteporfin

Verteporfin (VP) is used to treat classic subfoveal choroidal neovascularization due to age‐related macular degeneration. In a study by Barrette et al. [54], the ability of this Hippo pathway inhibitor to inhibit YAP–TEAD, a key mediator of tumor invasion and metastasis, was evaluated. In experiments, VP was found to effectively disrupt YAP/TAZ–TEAD signaling and alter transcriptional profiles associated with invasion, epithelial‐to‐mesenchymal transition, and proneural‐to‐mesenchymal transition, mimicking the effects of TEAD1 deletion. This action suppressed tumor cell migration and invasion in both primary and recurrent glioblastoma lines. In an aggressive orthotopic patient‐derived xenograft (PDX) model, short‐term administration of VP significantly reduced central and infiltrative tumor burden, accompanied by decreased expression of Ki67, nuclear YAP, TEAD1, and TEAD‐regulated targets, including EGFR, CDH2, and ITGB1. Long‐term VP treatment was also well tolerated and conferred a survival advantage in both PDX models, both as monotherapy in primary glioblastoma and in combination with temozolomide chemotherapy in relapsed glioblastoma. Interestingly, in combination with TMZ, VP treatment was also associated with increased MGMT promoter methylation [54].

Moreover, another study by Liu et al. employed three glioma cell lines (SF767, U373, and LN229) that exhibit distinct sensitivities to TMZ. Analyses revealed that TMZ responsiveness was positively associated with the expression levels of MGMT and the multidrug resistance transporter ABC subfamily G member 2 (ABCG2). Previous studies have shown that CK2 regulates MGMT and ABCG2 expression via STAT3 and Hippo–YAP signaling pathways, respectively. Based on this rationale, the CK2 inhibitor CX‐4945 and the YAP inhibitor verteporfin were evaluated in combination with TMZ. The results demonstrated that CX‐4945, but not verteporfin, enhanced TMZ sensitivity in the TMZ‐resistant SF767 cell line. Moreover, combined treatment with CX‐4945 and TMZ showed superior antitumor efficacy compared with TMZ monotherapy in glioma mouse models [121]. Thus, verteporfin may have antitumor and anti‐invasive activity in glioblastoma models, but its ability to enhance TMZ efficacy requires further validation.

## 18 Future Perspective

Initial preclinical and clinical findings have generated significant hope for repurposed drugs to enhance the efficacy of temozolomide (TMZ) in treating glioblastoma (GB). However, the pathway for translating these exciting results into standard clinical practice remains fraught with considerable obstacles. Glioblastoma, due to its profound molecular heterogeneity, does not respond uniformly to treatments; therefore, future studies must pave the way by focusing on patient stratification based on biomarkers. Precise identification of predictive biomarkers for drug sensitivity, such as MGMT methylation status, alterations in DNA repair pathways, or tumor metabolic characteristics, will provide a roadmap for selecting patients most likely to benefit from TMZ‐based therapeutic combinations. Integrating multi‐omics approaches with high‐throughput drug screening will be a significant step towards achieving truly personalized treatment strategies.

Although a multitude of repurposed drugs have demonstrated synergistic effects with TMZ in laboratory settings, only a small fraction are suitable for clinical translation due to factors such as toxicity profiles, pharmacokinetic properties, and, most importantly, the ability to cross the blood–brain barrier (BBB). Among these, drugs like Lomitapide and Nitroxoline, despite their theoretical potential, still lack sufficient studies regarding their BBB penetration. Future research must seriously focus on elucidating this critical aspect to determine whether these drugs can effectively reach the brain tumor. To identify the most promising combinations, it is necessary to develop advanced preclinical models that simulate the complex tumor microenvironment of GB. Models such as patient‐derived xenografts and organoids will serve as powerful tools for predicting clinical efficacy and selecting appropriate drug combinations for early‐stage trials. Furthermore, computational modeling and network‐based analyses can accelerate the rational selection of drug pairs with maximal therapeutic potential and minimal side effects.

Future clinical trials evaluating repurposed drugs in combination with TMZ should utilize adaptive trial designs. Coupled with real‐time biomarker monitoring, these approaches will enable rapid optimization of dosage, drug scheduling, and patient selection, thereby significantly increasing the chances of achieving meaningful clinical responses. Through deeper integration of mechanistic insights, rigorous preclinical validation, and precision‐guided clinical evaluation, repurposed drug‐based strategies hold the genuine potential to overcome resistance to TMZ and ultimately, substantially improve treatment outcomes for glioblastoma patients. Focusing on BBB penetration and identifying predictive biomarkers will be key steps in realizing this vision.

## 19 Conclusion

Cancer results from a series of cellular changes that lead to uncontrolled growth and resistance to programmed cell death. Understanding the key features of tumors provides a clear framework for identifying molecular pathways involved in cancer initiation, progression, and persistence, and for designing targeted, more precise, and less invasive therapies. In this context, drug repurposing, that is, the use of existing drugs for novel therapeutic purposes, has been considered a rapid, safe, and cost‐effective strategy compared to developing new drugs. This approach can accelerate the development of novel therapies to the clinic and, given the safety data background, reduce the likelihood of clinical failure. However, determining the optimal dose, overcoming pharmacokinetic and pharmacodynamic limitations, and meeting regulatory requirements remain major challenges for this strategy. Increasing evidence suggests that repurposed drugs can be effective in combination with TMZ in the treatment of glioblastoma. Drugs such as metformin, chloroquine, valproic acid, amlodipine, aspirin, and chlorpromazine act through diverse anticancer pathways and target multiple signaling pathways, highlighting their importance in combination therapy of GB. Importantly, the evidence is not uniformly positive for all repurposed agents; some combinations, including diazepam–TMZ and methadone–TMZ, have shown null, conflicting, or unfavorable results in preclinical studies, and further research is needed to translate these findings into the clinic. Key areas of future research include optimizing combination therapy strategies, identifying potential mechanisms of resistance, and validating efficacy in rigorous clinical trials. With further clinical validation, repurposed drugs could become an important part of glioblastoma treatment, providing new options for patients with limited treatment options.

## Author Contributions

**Amir R. Afshari:** investigation, writing – review and editing. **Mohammad Jalili‐Nik:** conceptualization, investigation, writing – review and editing, supervision. **Ali Nakhaei:** writing – original draft, investigation, conceptualization. **Farzaneh Davoudi:** writing – review and editing, investigation. **Elaheh Gheybi:** writing – review and editing, investigation. **Atefeh Taghavi:** writing – review and editing, writing – original draft.

## Funding

The authors have nothing to report.

## Consent

The authors have nothing to report.

## Conflicts of Interest

The authors declare no conflicts of interest.

## Data Availability

Data sharing not applicable to this article as no datasets were generated or analysed during the current study.
